# Economic Modeling of Heart Failure Telehealth Programs: When Do They Become Cost Saving?

**DOI:** 10.1155/2016/3289628

**Published:** 2016-07-26

**Authors:** Sheena Xin Liu, Rui Xiang, Charles Lagor, Nan Liu, Kathleen Sullivan

**Affiliations:** ^1^Philips Research North American, Briarcliff Manor, NY 10510, USA; ^2^Columbia University, New York, NY 10027, USA; ^3^Philips Home Health Services, Framingham, MA 01702, USA; ^4^Dignity Health, Santa Maria, CA 93458, USA

## Abstract

Telehealth programs for congestive heart failure have been shown to be clinically effective. This study assesses clinical and economic consequences of providing telehealth programs for CHF patients. A Markov model was developed and presented in the context of a home-based telehealth program on CHF. Incremental life expectancy, hospital admissions, and total healthcare costs were examined at periods ranging up to five years. One-way and two-way sensitivity analyses were also conducted on clinical performance parameters. The base case analysis yielded cost savings ranging from $2832 to $5499 and 0.03 to 0.04 life year gain per patient over a 1-year period. Applying telehealth solution to a low-risk cohort with no prior admission history would result in $2502 cost increase per person over the 1-year time frame with 0.01 life year gain. Sensitivity analyses demonstrated that the cost savings were most sensitive to patient risk, baseline cost of hospital admission, and the length-of-stay reduction ratio affected by the telehealth programs. In sum, telehealth programs can be cost saving for intermediate and high risk patients over a 1- to 5-year window. The results suggested the economic viability of telehealth programs for managing CHF patients and illustrated the importance of risk stratification in such programs.

## 1. Introduction

Congestive heart failure (CHF) is caused by any condition which reduces the efficiency of the heart muscle and results in insufficient blood supply to the human body. The high prevalence and costs associated with congestive heart failure (CHF) place an enormous economic and social burden on patients and society. Between 4 and 7 million people were estimated to suffer from CHF in the United States [1], with an estimated total direct and indirect cost of $39.2 billion in 2010 [2]. Over the last decade, the annual number of hospitalization incidences has increased from 0.8 to over 1.0 million for CHF as a primary diagnosis and from 2.4 to 3.6 million for CHF as a primary or secondary diagnosis over the last decade [3]. Around 50% of CHF patients were rehospitalized within 6 months of discharge; this trend is expected to continue to rise with an aging population [3, 4]. Hospitalization incidences are the most expensive events within the CHF care cycle, with admissions contributing 62.5% of total CHF medical costs [5]. Controlling the frequency and/or severity of exacerbation is of paramount importance to both quality of life of the patient and managing healthcare costs.

Telehealth is defined as the use of video, electronic, or other telecommunication information to monitor patients and transmit data related to patient health status at a distance [6]. Telehealth programs have been both theoretically and empirically proved clinically beneficial because deterioration can be quickly detected and addressed [7–9]. However, the current understanding of the cost consequences of these telehealth programs in the home care setting is still limited. Such disease management approaches could add costs over standard care due to their significant requirement for human and technical resources. However, they also bring about some level of cost savings through reduction of hospitalization incidences, length of stay (LOS), clinic visits, and the ancillary procedures. Understanding the cost-effectiveness of a CHF setting is the key motivation of the present work.

This study designs and applies a Markov model to assess long-term clinical outcomes and economic consequences of providing CHF telehealth programs. Costs are modeled, from the perspective of an American payer. The analysis includes telehealth install/uninstall costs, monthly monitoring costs, costs for nursing resources for data review and home visits, and pharmacy costs, as well as the usual CHF outpatient and inpatient cost. Scenario analysis was performed to assess clinically and economically feasible product performance-cost combinations. Through the model, we will be able to address the research questions of when and with whom the optimal cost saving can be achieved by deploying telehealth programs.

## 2. Methods

### 2.1. Telehealth Program for CHF Management

**Figure 1:**
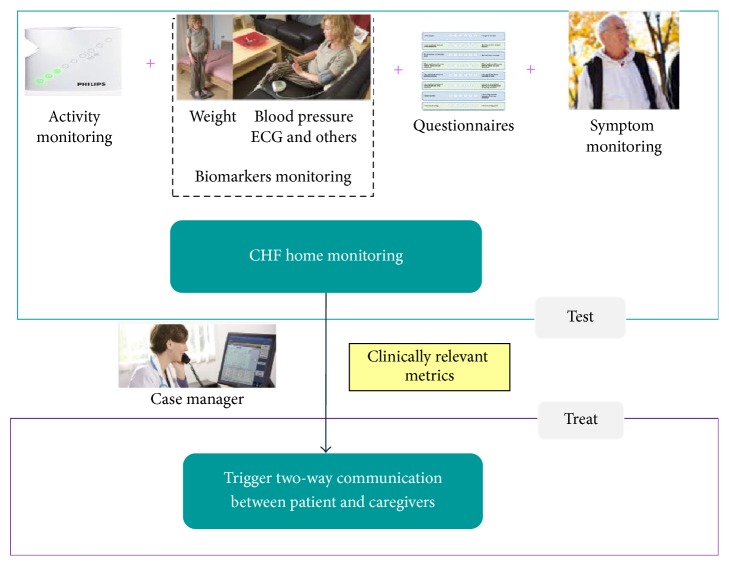
Schematic of a home-based telehealth program for monitoring CHF patients. Note that TEST includes one or more of the monitoring measures: activity monitoring, biomarker monitoring, questionnaires, and symptom monitoring; TREAT includes one or more of the following: case manager reviewing data, telephone triage, physicians' initiation of medication package, and nurse home visit (if needed).

The model was developed and is presented in the context of a home-based telehealth program on CHF. Telehealth programs can be conceptualized as having two primary components [10]: (1) TEST: monitoring and detection of possible deterioration; (2) TREAT: early treatment and intervention upon the detection of deterioration. Detailed descriptions of these components can be found in Figure 1.

### 2.2. Markov Model

**Figure 2:**
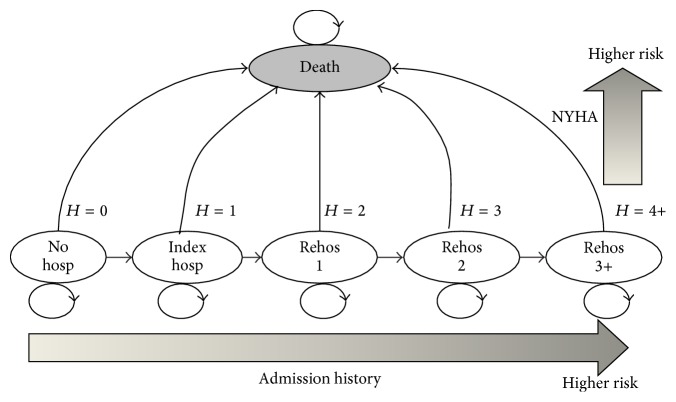
Markov model diagram.

Markov models are state transition models commonly used to estimate the cost-effectiveness of a new treatment [11]. They are most widely used in healthcare economics literature to model lengthy and recurrent situations like the progression of chronic diseases [12]. They have been previously employed to investigate different CHF interventions such as screening, pharmaceuticals, devices, and disease management programs [13]. The study implements a Markov model using rehospitalization as an indicator for disease progression. The patient population is comprised of 5 living states and a death state (see Figure 2). Living states are defined by the number of prior hospitalization incidences for CHF, an important predictor of disease progression and prognosis [14]. A patient is characterized in the model as belonging to one of these states. The risks of mortality and rehospitalization depend on New York Heart Association (NYHA) classes. For each monthly cycle, surviving patients may remain in the current state or at risk for hospitalization or death (either from CHF or non-CHF causes). The simulation is able to be carried out at any reasonable number of follow-up monthly cycles. Although most current telehealth programs are utilized for one-year time frame [7–9, 15], we examine the longer-term outcome by extending the time horizon of analysis for up to five years. Patient cohorts were analysed using this Markov model under both usual care (nontelehealth) and telehealth scenario (hosp/H: hospitalization; Rehos: Rehospitalization).

### 2.3. Risks in Hospitalization and Mortality in Usual Care

The usual care cohort is defined as the cohort without receiving any telehealth intervention. The risks of hospitalization and mortality for the usual care group were derived from previous models [11, 18] and their associated trial data [16, 17].  Table 1 summarizes the transition probabilities used in the current model. At the start of each monthly simulation cycle, patients move to different states according to these transition probabilities for hospitalization and death.

### 2.4. Efficacy of Telehealth Intervention

The mortality and hospitalization risks for patients in the telehealth group are affected by telehealth program efficacy. These risks are estimated in our previously published meta-analysis performed on 33 randomized control trials (RCT) between 2001 and 2012 from more than 9 countries with a total of 7530+ patients [15]. Follow-up of the individual studies varied with a median duration of 12 months. Key results for the meta-analysis are given in Table 2. In the current model, these meta-analysis findings were used to adjust the transitional probabilities for telehealth group through proportional reductions in transitional probabilities of usual care group as described in Table 2. Due to lack of patient level data, the model assumes constant reduction effectiveness across all cycles for telehealth groups.

### 2.5. Resource Use and Cost Data

All costs were fixed at 2013 US dollars ($) for the duration of the study period and final costs are represented in nominal dollars. Costs were calculated for each group from the perspective of American public payers. Indirect costs such as loss of productivity and the increase in sick days were not assessed as we focused solely on direct healthcare costs.

Our models synthesized inpatient and outpatient contributions to both CHF and non-CHF healthcare costs based on previously published studies. The cost estimates are summarized in Table 3. Besides usual care costs, the telehealth intervention cohort incurred additional costs. The recurring cost of the telehealth program (which is beyond the cost of usual care) consists of (1) the cost of monthly monitoring or TEST cost and (2) cost of early treatment when the monitoring gives a positive results or TREAT cost. These additional costs were estimated from field experts.

**Figure 3:**
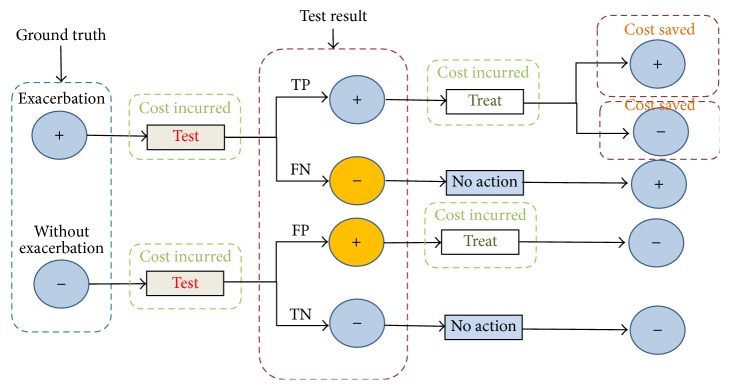
Cost consequences of deploying telehealth programs with certain exacerbation detection sensitivity and specificity. Note that there are two cost saving channels: when true exacerbation (+) is converted to nonexacerbation status (−), cost is saved through reverted admission. Even when true exacerbation is not reverted, through telehealth monitoring and early intervention, the severity of exacerbation can be reduced such that even if the patients are admitted to hospital, the length of stay would be reduced.

The costs of telehealth programs are affected by two additional technical parameters: (1) the sensitivity of the home-based exacerbation detection method (SEN) and (2) the specificity of the home-based exacerbation detection (SPE) [10]. Sensitivity refers to the probability of a positive test in a patient with an acute onset of CHF. Specificity refers to the probability of a negative test in a patient without an acute onset of CHF. These can be written as SEN = TP/(TP + FN) and SPE = TN/(TN + FP), where TP is true positive (true exacerbation and the test is positive), FN is false negative (has exacerbation but not detected), TN is true negative (no exacerbation and the test is negative), and FP is false positive (the test is positive but no exacerbation). Note that a FP will cause unneeded home-based treatment and thus incur unnecessary cost; a FN, or missed diagnosis, will omit patients from the early treatment and thus will not reduce admissions but still incur monitoring expenditures.  Figure 3 provides an explanation of sensitivity and specificity and their relationship to the cost change in the telehealth program.

### 2.6. Outcomes

The three main outcomes of the model are the number of incremental hospitalization incidences, incremental health outcome, and total cost difference. Health outcome is expressed as life years (LY). Utility values per disease state are not considered in this study. All costs were discounted at a rate of 3.0%, an accepted value for the United States [23].

### 2.7. Base Case

Three pairs of cohorts, each consisting of a telehealth cohort and a usual care cohort, were constructed. Within all cohorts, patients were distributed in the NYHA II or III population. Cohort 1 (C1) begins at the time when patients have no hospitalization at all. This cohort indicates the lowest risk population of heart failure. Cohort 2 (C2) initially contains a 30%, 30%, and 40% distribution of patients with one, two, and three prior hospitalization incidences, respectively. This cohort resembles the clinical cohort of patients with middle-to-high risk who are also considered as the target population for current-day telehealth programs. Cohort 3 (C3) is composed entirely of patients who have already had at least four prior CHF hospital admissions. This cohort represents severe, very advanced patient population whose condition deteriorates fast and is subject to frequent hospital admissions. All cohorts were tracked through Markov cohort analysis over the five-year simulation horizon. First-year, third-year, and fifth-year results were recorded, and overall outcomes were estimated at these time points.

### 2.8. Sensitivity Analysis

We additionally performed both one-way and two-way sensitivity analyses to investigate the effect of adjusting base case assumptions such as costs and transitional probabilities. Three scenarios for the performance of telehealth program were constructed to evaluate the impact of changing the telehealth efficacy parameters. Each scenario was defined by a different combination of five parameters as described in in Table 4, representing a base case, a best case, and a worst case performance scenario. Markov analyses were again executed in each of these three scenarios for all three cohort pairs. The base case parameters for modeling telehealth efficacy are given in Table 4 as well.

## 3. Results

### 3.1. Validation

We validated our model using the lifetime cost of the control arm, that is, the usual care cost of heart failure. Dunlay et al. estimated that total lifetime costs after heart failure diagnosis were $109,541 (95% confidence interval, $100,335 to 118,946) per person in 2008 dollars [24]. We simulated a cohort of the newly diagnosed CHF population (i.e., cohort 1, where no patients had any prior admissions) and investigated the lifetime cost by using 20-year time horizon (when over 95% of all patients have died). The total predicted cost from our model is $114,939 per patient.

Furthermore, we estimated the current economic burden of CHF in the United States by constructing a cohort where the weights of admission status were derived from the real statistics of American patient population (i.e., 70.7%, 10.3%, 4.4%, 3.3%, or 11.3% with 0, 1, 2, 3, or 4 or more admissions, resp., [11, 25]). With 5 million affected CHF cases, the reported yearly direct costs of CHF in the states are between $33.7 billion [26] and $39.3 billion [27]. The model estimated yearly direct cost of American CHF is $36.2 billion which is in line with the reported value derived from multiple claim database analysis (clinical trials or population studies) conducted by existing literatures [26, 27].

### 3.2. Base Case Analysis

**Figure 4:**
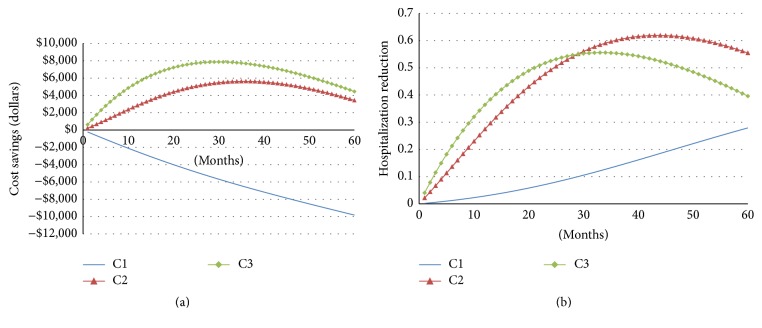
Base case analyses for three cohorts. (a) Cost saving curves as a function of number of years on telehealth programs; (b) hospitalization reduction curves as a function of number of years on telehealth programs.

Results for the three hypothetical cohorts are given in Table 5. The base case analysis yielded cost savings ranging from $2832 (intermediate risk cohort C2) to $5499 (high risk cohort C3) and 0.03- to 0.04-life year gain per patient over a 1-year period. Applying telehealth solution to a low-risk cohort with no prior admission history would result in $2502 cost increase per person over the 1-year time frame with 0.01-life year gain. Expanding over a 3-year time frame, applying the telehealth solution to low-risk cohort (C1) would result in $6590 cost increase per person 0.08-life year gain. For the intermediate risk (C2) and high risk (C3) cohorts, the cost of telemonitoring is entirely offset through reduced hospital utilization, with additional cost savings of $5620 and $7683 per person, as reported in Table 5 (shown in years) and depicted in Figure 4 (shown in months).

### 3.3. Sensitivity Analysis and Scenario Analysis

The focus of the sensitivity analysis was to examine how different assumptions on telehealth efficacy would impact the estimated costs and clinical outcomes. We use cohort 2 in this analysis as this cohort represents the most clinically realistic population who might benefit most from telehealth programs.

**Figure 5:**
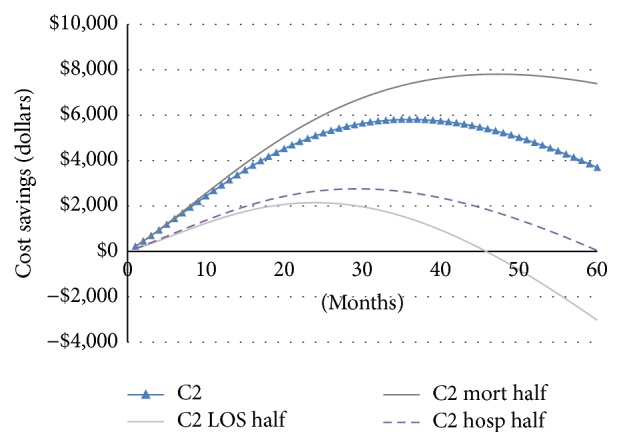
Sensitivity analysis of cost saving curve for cohort 2.

In one-way sensitivity analysis, we reduced the default efficacy from full capacity (base case from meta-analysis) to 50% effectiveness. As indicated in Figure 5, the cost saving capacity of telehealth is more sensitive to LOS reduction than hospitalization reduction. Mortality reduction has the opposite effect: the less the mortality reduction is, the more cost savings it would bring about as more people would live longer and consume more costs, without considering end-of-life care, transplants, VADs, or any other enormously costly final options.

Two-way sensitivity analysis was performed to investigate the effect of adjusting admission costs (from $6000 to $16000) and the monthly telehealth TEST cost (from $50 to $450), under the assumption of default telehealth efficacy (base case from meta-analysis). For example, if admission costs are $10,000 and monthly telehealth fee is $250, applying telehealth programs can save $2,109 in total in a three-year period by reducing the admission rates according to our model (see Table 6).

Moreover, the base case analysis assumes that patients at different risk levels consume the same amount of telehealth services (TEST cost is $220 per month, see Table 3). It is reasonable to argue that high risk patients would benefit more from active monitoring (which involves hardware data recording and transmission) than low-risk patients, resulting in a higher TEST cost for those patients. For low-risk patients, less intensive and less costly telehealth services (such as coaching and consultation) may be sufficient. A sensitivity analysis was performed to identify the maximum monthly TEST cost to be cost saving in the given time horizon assuming no changes in clinical efficacy from these varying levels of service.

In the context of a cost-avoidance model, the break-even point was defined as the cost for which the total cumulative telehealth costs for the CHF patients equalled the total cost saving through hospitalization and LOS reduction (cost savings or ΔCost = 0). Results for this break-even analysis are given in Table 7.

## 4. Discussion

In this paper, we apply Markov methods for examining the potential cost consequences of home-based telehealth programs that attempt to reduce the frequency and severity of exacerbations in CHF. We investigated multiple scenarios for cost and clinical performance for the program and assessed the potential cost-saving capabilities of these programs from the perspective of an American payer. Through these analyses, we demonstrated the likely cost-saving capabilities of the CHF telehealth program and report on the technical and cost boundaries within which the program should operate.

Using meta-analysis results compiled over a broad range of clinical trials on CHF telehealth programs, we were able to define base case assumptions and scenarios. Our analysis suggests that, under the base case system performance and cost assumptions, telehealth programs are likely to be cost saving for higher risk patients (patients with one or more prior admissions) within the simulation duration (up to five years).

To our knowledge, this study is the first of its kind to assess telehealth economic and clinical consequences in chronic heart failure. The base case analysis yields cost savings ranging from $2832 (cohort 2) to $5499 (cohort 3) per patient over a 1-year period. The cost saving capacity of telehealth is most sensitive to the baseline cost of hospital admission and the LOS reduction ratio by telehealth programs. This result suggests that regions with high costs of inpatient care for CHF and high readmission rate would receive the greatest financial benefit from telehealth programs.

We chose a 5-year period as the longest observation period for analysis because most current-day telehealth programs were used for 6 months (33%) and 12 months (51.5%). Only 6% studies extended over 24-month time frame [15]. Our study indicates that cost savings from telehealth rise to a peak at around 2 to 3 years and then start to decline over time. The trend of parabolic curve is because the survival of the telehealth group is higher than that of the usual care group, resulting in long-term survivors continuing to incur greater costs over time. It was projected that the cost saving would continue to decline toward zero, that is, becoming cost-incurring in the long run. The result of long-term cost-incurring is consistent with the results of Chan et al. (2008) who found that the managed care programs cost $9700 per life year gain in the base case by following patients for 15 years [11]. Göhler et al. (2008) simulated the lifetime managed care programs in CHF patients and indicated that this number would be €8900 per quality-adjusted life year gain [18].

This study had a few limitations: first we did not include utility data into the analysis; second we obtained model data from existing literatures and assume that the effectiveness of telehealth programs is constant over time. Future work could include patient level data when available and create time-dependent transition probabilities.

We envision that the results of this study and the broad approach can aid payers in technology acquisition decisions. We also suggest that the results of this study can be used to set performance and price targets for those healthcare innovators engaged in the development of CHF telehealth programs. Payers could use the model developed in this study to simulate different scenarios that would help them assess how to best allocate telehealth resources among different patient risk. For example, payers can evaluate if the intensities and cost of the teleheath intervention are reasonable given the patient risk profile and if the cost impact of the intervention is satisfactory according to their perspectives.

## Figures and Tables

**Table 1:** Probability of mortality and hospitalization.

Usual care	Definitions	NYHA II or III	NYHA III or IV
*Probability*			

Death rate			
*Not hospitalized*		0.007 [11, 16]	0.01 (0.01–0.015 [17])
*Hospitalized*		0.100 (0.07–0.1 [18])	0.100 (0.07–0.1 [17, 18])
Hospitalization			
*H* = 0	No prior hospitalization	0.008 [11, 16]	0.008 [17, 18]
*H* = 1	Index admission	0.052 [11, 16]	0.168 [17, 18]
*H* = 2	2 previous admissions	0.106 [11, 16]	0.213 [17, 18]
*H* = 3	3 previous admissions	0.121 [11, 16]	0.268 [17, 18]
*H* = 4*+*	4+ previous admissions	0.180 [11, 16]	0.334 [17, 18]

**Table 2:** Reduction effectiveness of different types of telehealth programs [15].

	Measure	Models	Effect	95% CI	*P* value	Heterogeneity (*I* ^2^ test)	*Q* (*P*)	Public bias	Effectiveness
Mortality	RR	FE	0.76	(0.66, 0.88)	<0.001	18.3%^*∗*^	25.4 (0.49)^+^	No	24% reduction
CHF hosp	RR	RE	0.72	(0.61, 0.85)	<0.001	66.3%^*∗*^	61.8 (<0.001)^++^	No	28% reduction
CHF LOS	MD	RE	−1.41	(−2.43, −0.39)	0.007	71.3%^*∗*^	38.6 (<0.001)^++^	No	1.41-day reduction

RR: risk ratio; MD: mean difference.

FE: fixed effect model. RE: random effect model.

^*∗*^ *I* squared < 20% indicated small heterogeneity; *I* squared > 20% indicated high heterogeneity.

^+^ *Q* test was not significant so that no significant heterogeneity among studies was presented; fixed effect (FE) model was able to be used.

^++^ *Q* test was significant so that there was significant heterogeneity among studies; random effect (RE) model had to be used.

**Table 3:** Cost estimates.

	Baseline	Quoted references
Usual care		
Per CHF hospitalization cost	$12,000	$12.7K [19], $11K [20], 10.9K [21], 12K–18K [22]
Annual CHF outpatient cost	$1,700	$680–2700 [11]
Annual non-CHF healthcare cost	$10,000	$7300–13000 [11]
Telehealth		
Install/uninstall cost amortized to each month	$15	Based on field experts estimate
Monthly monitoring cost	$80	Based on field experts estimate
Case manager cost per patient per month	$125	Based on average nurse salary, assuming 75 patients are covered by one nurse
Total monthly TEST cost	$220	
Physician contact/medication initialization cost per detected episode	$52	Based on physician verbal order time and new medication cost
Nurse home visit cost per detected episode	$135	Based on field experts estimate
Total TREAT cost per episode	$187	

**Table 4:** Telehealth clinical efficacy parameters.

	Best scenario	Base case scenario [15]	Worst scenario
Sensitivity	90%	80%	70%
Specificity	90%	80%	70%
Mortality reduction	29%	24%	19%
Hospitalization reduction	38%	28%	18%
LOS reduction	30%	25%	20%

**Table 5:** Base case results.

			Year 1			Year 3			Year 5		
			Cost	LF	AD	Cost	LF	AD	Cost	LF	AD
C1	Low risk	Usual	12402	0.94	0.11	34982	2.47	0.48	54780	3.63	1.00
		Tele^†^	+2502	+0.01	−0.02	+6590	+0.08	−0.14	+9826	+0.21	−0.28

C2	Intermediate risk	Usual	25304	0.88	1.23	66812	2.07	3.51	93075	2.74	5.03
		Tele^†^	−2832	+0.03	−0.27	−5620	+0.22	−0.60	−3422	+0.46	−0.55

C3	High risk	Usual	32916	0.84	1.90	75515	1.91	4.39	99024	2.47	5.79
		Tele^†^	−5499	+0.04	−0.36	−7683	+0.25	−0.55	−4456	+0.50	−0.4

AD: admission; LY: life years.

^†^Telehealth results are incremental values, compared to usual care.

**Table 6:** Two-way sensitivity analysis: telehealth 3-year incremental cost and cost-effectiveness with varying monthly telehealth service costs and hospital admission costs.

Telehealth monthly cost ($)	Admission cost ($)					
	6K	8K	10K	12K	14K	16K
	Δ(cost)^*∗*^	Δ(cost)	Δ(cost)	Δ(cost)	Δ(cost)	Δ(cost)
50	−2609	−5264	−7920	−10575	−13230	−15886
150	295	−2359	−5014	−7670	−10325	−12980
250	3201	546	−2109	−4764	−7419	−10075
350	6106	3451	796	−1858	−4514	−7169
450	9012	6357	3701	1046	−1608	−4264

^*∗*^Negative delta cost indicates cost saving.

**Table 7:** Break-even costs for different patient risk groups to reach cost saving in 1, 3, and 5 years.

Patient group		Maximum monthly service fee ($)								
		Best			Base case			Worst		
		Year 1	Year 3	Year 5	Year 1	Year 3	Year 5	Year 1	Year 3	Year 5
C1	Low risk	$35	$48	$56	Never	$16	$23	Never	Never	Never
C2:	Intermediate risk	$634	$552	$404	$472	$414	$303	$313	$277	$204
C3	High risk	$946	$652	$430	$715	$498	$333	$497	$349	$236

## Additional Points

(i) Telehealth programs have been both theoretically and empirically proved clinically beneficial, but current understanding of the cost consequences of these telehealth programs is still limited. (ii) This study develops a Markov model and assesses clinical and economic consequences of providing telehealth programs for CHF patients. This is the first attempt in this field. (iii) Telehealth programs can be cost saving for intermediate and high risk patients with one or more prior admissions over a 1- to 5-year window. The cost savings were most sensitive to patient risk, baseline cost of hospital admission, and the length-of-stay reduction ratio affected by the telehealth programs. Regions with high inpatient care costs and high readmission rate would receive the greatest financial benefit from telehealth programs.
